# Safeguarding Open Science from exploitative practices

**DOI:** 10.1371/journal.pmed.1004851

**Published:** 2025-12-11

**Authors:** Danny Maupin, Matt Spick, Nophar Geifman

**Affiliations:** School of Health Sciences, Faculty of Health and Medical Sciences, University of Surrey, Guildford, Surrey, United Kingdom

## Abstract

Open research and data transparency are a bulwark against unethical activities, but can also introduce integrity risks. In this Perspective, Danny Maupin, Matt Spick and Nophar Geifman argue that freely available data can be exploited, and set out the case for the use of safeguarding practices.

## Teaser

Open research and data transparency are a bulwark against unethical activities, but can also introduce integrity risks. As with all public goods, freely available data can be exploited, and here we set out the case for the use of safeguarding practices.

The advent of Generative Artificial Intelligence (GenAI) and its ability to create fake data, images and text represents an unprecedented challenge to the integrity of the scientific literature. Data transparency through Open Science acts as a crucial safeguard against such fraudulent or unethical activity, providing an auditable trail of evidence [1]. This is in addition to other Open Science benefits, such as equitable data access and improved reproducibility of research [2]. There is, however, a fundamental and circular problem with making scientific data freely available as a defence against AI-generated fraud or other unethical behaviours: Datasets are themselves the fuel for AI engines. In other words, the very measures designed to fight fraud (open access) simultaneously power new forms of problematic activities (GenAI-assisted fast churn science). Robust evidence is emerging that open access datasets, especially in health and medicine, are being exploited in this way by paper mills and other bad actors [3,4].

**Fig 1:**
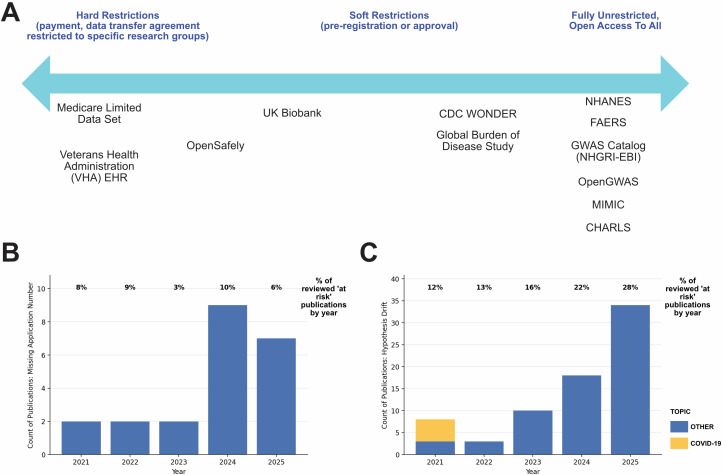
The safeguarding spectrum of public health datasets. **(A)** Datasets by access type. **(B)** Publications using UK Biobank data without an application number. **(C)** Publications using UK Biobank data with hypothesis drift vs. original research goals on UK Biobank website. % for 2021 excludes COVID-19 related publications.

High volumes of low-value or outright misleading research have many undesirable consequences including misallocation of funding, distortion of assessment metrics and reduced trust in the scientific literature. Both publishers and the wider scientific community are taking action in areas such as fake or duplicated images, the identification of tortured phrases used to avoid plagiarism checks [5], or other signals of paper mill activity; albeit this is an adversarial process and problematic research is still being published [6,7]. Nevertheless, the policies of data providers are still mostly seen through the prism of Open Science and a desire to have as much data available to as many people as possible. While some datasets operate a system of controlled access, others, e.g., the Centre for Disease Control’s National Health and Nutrition Examination Survey (NHANES), are fully open access, AI-ready and easily exploited (**Fig 1A**) [8]. Although for sensitive information (e.g., personal data that cannot be anonymized) a controlled system is clearly required, the treatment of other data types is more contested. We argue that unfettered open access is undesirable and leads to unwanted behaviours including choosing methods and data to create the illusion of statistical significance (p-hacking), hypothesising after the results are known (HARKing) and the introduction of false discoveries to the literature [9]. Conversely, heavily restricted closed systems are inequitable, and run the risk of being monopolised by users with specific research objectives or biases towards particular viewpoints on health issues.

Other resources have taken a middle path (Fig 1A), for example, the UK Biobank, which provides access to de-identified data for all eligible researchers globally—including academics, charities, and commercial entities—provided the research is health-related and in the public interest. This is managed through the Access Management System, which includes reviews by a committee to ensure proposals align with its ethical framework and public interest mandate. Once approved and a legal transfer agreement is signed, researchers are granted access to the data, typically via the secure, cloud-based UK Biobank Research Analysis Platform. A core condition of access is a prohibition on attempting to identify participants; researchers additionally pay an access fee, return their results to the Biobank for the benefit of future research, and are obliged to acknowledge their use of UK Biobank data and their application number in all published research.

This type of approach has many advantages. The UK Biobank’s Access Management System and the use of application-specific registration IDs act as a form of pre-registration (as these are reported openly on the UK Biobank website), protecting against ‘salami slicing’ (slicing one set of results across multiple papers) and HARKing. Such requirements are, however, only as effective as their enforcement. A pilot audit of 321 research papers published between 2021 and 2025, conducted as part of a pre-registered protocol [10] using UK Biobank data targeted at topics previously identified as featured in formulaic/low-quality research templates, found that 7% did not report or appear to have a valid UK Biobank application number (Fig 1B). 25% exhibited substantial hypothesis drift compared with the disclosed research goals on the UK Biobank website. The proportion of publications without an application number varied by year but did not show any clear trend, but the proportion showing hypothesis drift increased each year, from 12% in 2021 to 28% in 2025 (Fig 1C). This may also reflect growth in post-application waivers by the UK Biobank, such as likely happened in 2021 with COVID-19-focussed research, but these are not disclosed. Whilst adding new projects to existing applications is convenient, we argue that—without public disclosure of approved variations—this reduces the effectiveness of publishing approved research questions as a form of integrity control and potentially enables fast-churn science and HARKing.

While these findings are derived from a purposive sample and cannot be extrapolated to the whole body of UK Biobank-derived research, they demonstrate the vulnerability of open-access data sources. Here, we are using the UK Biobank as a case study only, and would not expect it to be more compromised than any other data source; indeed, our contention is that all open-access data sources are public goods that will be exploited by unethical actors. Those arguing for open and equitable access to research data make their case based on strong ethical arguments. In addition, in experimental research, open access supports reproducibility and has so far been less susceptible to ‘fast churn science’. Once a dataset is compromised and credibility has been damaged, however, it becomes more challenging for researchers to publish research. NHANES provides an illustration of this phenomenon, with some publishers no longer accepting submissions based on open-access public health datasets [11]. In other words, there is a real cost to not exerting some measure of preventive control over data usage.

Of course, data providers are not enforcement agencies for good science. Historically, a trust-based system has worked well, but GenAI allows unethical actors to achieve higher productivity than ethical researchers and in our view, has enabled the explosion in formulaic manuscript production since 2023. We suggest that unrestricted open access will continue to compromise trust in research using exploited assets, and that safeguarding measures will only be truly effective with better disclosure processes (for example, disclosing variations to approved research questions) and publication checks. The latter could include de-registering applications that show signs of significant hypothesis drift and an increased focus on whether data have been obtained legitimately, in line with Committee on Publishing Ethics guidance on authorised data use [12]. These safeguards would not be incompatible with Open Science, but would protect open practices and maintain the ethical and scientific benefits; without such changes to reassert the balance between Open Science and research integrity, our expectation is that the credibility of research based on open access data sources will continue to decline.

## Abbreviations

GenAI: Generative Artificial Intelligence
HARKing: hypothesising after the results are known
NHANES: National Health and Nutrition Examination Survey
